# Conceptualising production, productivity and technology in pharmacy practice: a novel framework for policy, education and research

**DOI:** 10.1186/s12960-018-0317-5

**Published:** 2018-10-03

**Authors:** D Baines, I Bates, L Bader, C Hale, P Schneider

**Affiliations:** 1 Warwick Medical School, University of Warwick, Coventry, CV4 7AL United Kingdom; 2 FIP-UCL Collaborating Centre, UCL School Pharmacy, 29-039 Brunswick square, London, WC1N 1AX United Kingdom; 3 Bournemouth University, Fern Barrow, Poole, Dorset BH12 5BB United Kingdom; 4 University of Arizona, Colleges of Pharmacy and Public Health, Phoenix Biomedical Campus, 650 East Van Buren Street, Phoenix, AZ 85004-2222 United States of America

## Abstract

**Context and background:**

People and health systems worldwide face serious challenges due to shifting disease demographics, rising population demands and weaknesses in healthcare provision, including capacity shortages and lack of impact of healthcare services. These multiple challenges, linked with the global push to achieve universal health coverage, have made apparent the importance of investing in workforce development to improve population health and economic well-being. In relation to medicines, health systems face challenges in terms of access to needed medicines, optimising medicines use and reducing risk.

In 2017, the International Pharmaceutical Federation (FIP) published global policy on workforce development (‘the Nanjing Statements’) that describe an envisioned future for professional education and training. The documents make clear that expanding the pharmacy workforce benefits patients, and continually improving education and training produces better clinical outcomes.

**Aims and purpose:**

The opportunities for harnessing new technologies in pharmacy practice have been relatively ignored. This paper presents a conceptual framework for analysing production methods, productivity and technology in pharmacy practice that differentiates between dispensing and pharmaceutical care services. We outline a framework that may be employed to study the relationship between pharmacy practice and productivity, shaped by educational and technological inputs.

**Method and results:**

The analysis is performed from the point of view of health systems economics. In relation to pharmaceutical care (patient-oriented practice), pharmacists are service providers; however, their primary purpose is not to deliver consultations, but to maximise the quantum of health gain they secure. Our analysis demonstrates that ‘technology shock’ is clearly beneficial compared with orthodox notions of productivity or incremental gain implementations. Additionally, the whole process of providing professional services using ‘pharmaceutical care technologies’ is governed by local institutional frames, suggesting that activities may be structured differently in different places and countries.

**Discussion and Conclusion:**

Addressing problems with medication use with the development of a pharmaceutical workforce that is sufficient in quantity and competence is a long-term issue. As a result of this analysis, there emerges a challenge about the profession’s relationship with existing and emerging technical innovations. Our novel framework is designed to facilitate policy, education and research by providing an analytical approach to service delivery. By using this approach, the profession could develop examples of good practice in both developed and developing countries worldwide.

## Background

People and health systems worldwide face serious challenges due to shifting disease demographics, rising population demands and weaknesses in healthcare provision [1]. Health problems are further exacerbated by current and projected global short-falls in health workers, especially in developing nations [2]. These multiple challenges, linked with the global push to achieve universal health coverage and the United Nations Sustainable Development Goals, have made apparent the importance of investing in workforce development to improve population health and economic well-being [3, 4]. In relation to pharmaceuticals, health systems face challenges in terms of (i) guaranteeing access to needed drugs [5], (ii) rationalising medicines use [6] and (iii) avoiding harm from adverse events [7]. For medicine systems to be strengthened, pharmaceutical education needs constant research, development and evaluation. There is a pressing need to better understand the relationships between production, productivity and technology in pharmacy practice to inform professional policy, education and research.

### Improving patient outcomes

In 2017, FIP published 67 statements on Pharmacy and Pharmaceutical Sciences Education (‘the Nanjing Statements’) that describe an envisioned future for professional education and training [8]. Underpinning these statements are the beliefs that (i) expanding the pharmaceutical workforce benefits patients and (ii) continually improving education and training produces better clinical results. This paper responds to these beliefs by outlining a framework that may be employed to study the relationship between pharmacy practice and productivity, which is shaped by educational and technological inputs. In suggesting our approach, we make the following key assumptions. First, there is a difference between ‘efficacy’ and ‘effectiveness’ in the sense that products that prove efficacious in clinical trials may differ in their effectiveness when used in practice [9]. Closing or narrowing the gap between efficacy and effectiveness may provide more value from the use of medicines [9]. In addition, there is support for the argument that gaps resulting from drug-related problems—whether clinical [10, 11] or economic [12–15]—can be identified, prevented and solved by pharmacists through the provision of pharmaceutical care [16].

Next, we assume that pharmacists can improve patient outcomes by reducing the occurrence of adverse events. For instance, pharmacy interventions can help reduce medical errors amongst older patients. Finally, we assume that pharmacists have a role in understanding and managing the unintended consequences of medicines use, which may also extend to handling the unwanted effects of the adoption of novel technologies [17, 18]. Pharmacists are well-equipped to reduce the occurrence of such side effects and to optimise medicines use. With these assumptions in mind, we have developed a conceptual framework for analysing the relationship between pharmacy practice and productivity.

### Production and productivity

Currently, the pharmacy profession performs two distinct types of activities: (i) medicine access and supply and (ii) pharmaceutical care. As a form of production, dispensing is an organisational process (fed by the physical inputs of capital, labour and consumables) that responds to prescribers by supplying requested medicines; prescribing is the most common point in the system of medicine use where errors occur, in both acute and ambulatory care settings [10, 11]. In contrast, pharmaceutical care involves decision-making about medicines therapy and planned consultations between pharmacists, prescribers and patients that facilitate the aim of improving health outcomes [19]. This paper presents a conceptual framework that represents the two production processes and makes suggestions for the conceptualisation of ‘productivity’ in each.

## Methods

### Professional vision

Pharmaceutical care was first envisaged as a professional service offering [20]. By re-focusing, re-educating and re-orientating, pharmacists would evolve into clinicians, working face-to-face with patients and collaborating with physicians on behalf of the patient. This way of thinking evolved during the 1980s and 1990s, when the philosophies of ‘knowledge-management’ and ‘portfolio-working’ encouraged professionals in many service industries to develop their knowledge and skills to work in partnership with many different clients, often on a fee-for-service basis. Whilst the ideological environment encouraged pharmacists to re-professionalise, the rest of the world was responding to the computer revolution [21]. As a result, the opportunities for harnessing new technologies in pharmacy practice have been relatively ignored. There is a clear vision for pharmaceutical care, but less clarity about the profession’s relationship with existing and emerging technical innovations. This paper aims to help redress this imbalance by providing a novel framework for analysing the relationship between pharmacy practice and the innovative dispensing and pharmaceutical care technologies, which may be used by policy-makers, researchers and educators in the field.

### Current literature

There is a small, but constant stream of published studies that evaluate the impact of new technologies on pharmacy practice and patient outcomes. We suggest that pharmacy technologies be divided along the following lines, using classifications based upon the foundational work of Goundrey-Smith. ‘Prescribing-dispensing technologies’ include electronic health records, electronic prescribing, the electronic transfer of prescriptions, robotics and barcode dispensing. ‘Pharmaceutical care technologies’ include mobile health, telecare, monitoring technologies and smart pumps. There is currently a need for scoping reviews of the literature in these two groups. An initial analysis suggests that most published studies in these areas are either quantitative or qualitative evaluations that test specific technologies in a pharmacy-related setting. As a body of work, current studies fail to (i) model technology as an integral feature of pharmacy practice, (ii) link new technologies with production processes in dispensing and pharmaceutical care and (iii) explain how technological advances affect productivity in drug supply and patient service provision. Given these failings, there is a need for a novel framework that helps policy-makers, educators and researchers conceptualise the relationships between production, productivity and technology in pharmacy practice. This framework must be applicable internationally across varying health care systems.

## Results

### Proposed framework

The purpose of this paper is to present a conceptual framework for analysing production methods, productivity and technology in pharmacy practice that differentiates between dispensing and pharmaceutical care services. The analysis is performed from the point of view of health systems economics. Because the fundamentals of pharmacy practice are analysed, our approach is applicable internationally. ‘Plumbing diagrams’ are presented in the sections below that show the main connections between key elements in the processes for dispensing and pharmaceutical care. The diagrams are designed to enhance our understanding of the structure of pharmacy practice. To achieve the aims of the paper, we perform the following tasks: First, we outline two heuristic models that show the different processes employed for dispensing and pharmaceutical care. Next, economic analysis is used to represent the relationship between labour inputs and pharmacy practice outputs and to demonstrate how a ‘technology shock’ can spur improvement in levels of productivity. Third, we present separate models for automated dispensing and technology-enabled pharmacy, as a means of showing how different technologies can influence professional practice. Fourth, we discuss the relationship between education and technology and suggest that the future may lie with ‘blended pharmacy practice’. Finally, we make recommendations for further research and policy.

## Production

‘Production’ may be defined as ‘the means of creating specified outputs with specific inputs’. In the context of pharmacy practice, two production processes generally employed are (i) the supply and dispensing of requested medicines and (ii) the generation of health-related pharmaceutical care outputs. The sub-sections below outline formal models for both.

### Drug dispensing

**Fig. 1:**
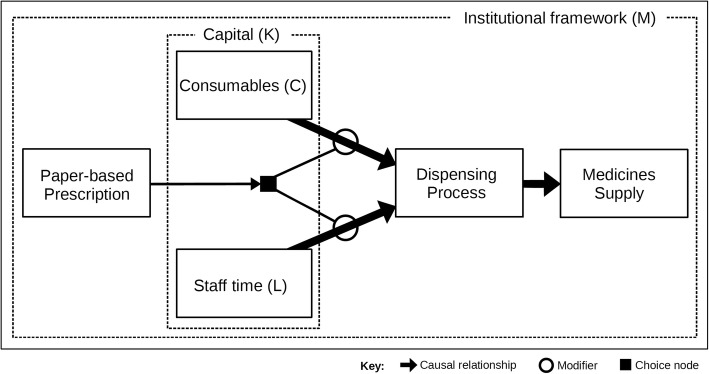
Manual dispensing process

Dispensing is a physical supply process. The relationship between inputs and outputs is normally stable, predictable and easily controlled. Figure 1 is a conceptual representation of the typical dispensing process. Demand, usually in the form of paper-based prescriptions written by practitioners, is shown on the left of the diagram. The arrival of a prescription triggers a choice (shown by the black square) to employ consumables (that is, the items used during dispensing, including computing, labelling, packaging and container costs) and staff time (in terms of pharmacists and technicians) to operate the dispensing process, which results in the supply of the requested medicine. The diagram also shows that the use of labour (L) and consumables (C) depends upon capital (K) expenditure because levels of investment (for instance, in barcode dispensing) affect how staff work and associated dispensing costs. Throughout our framework, L refers to any labour inputs used in the production process and includes pharmacists, technicians and other auxiliary staff. Finally, the dotted box around the edges of Fig. 1 implies that individual dispensing processes are influenced by the ‘institutional framework’ relevant to their local pharmacy practice jurisdiction. An ‘institution’ may be defined as the ‘law, regulations, policy, rules, conventions, ethics and norms governing behaviour’ [22]. In most countries, the institutions governing dispensing are clearly defined in national law and professional codes of conduct. For instance, the requirement that a pharmacist be present when medicines are supplied will limit the ways in which production may be organised. Therefore, dispensing processes may differ between places with different institutional frames [23].

### Pharmaceutical care

**Fig. 2:**
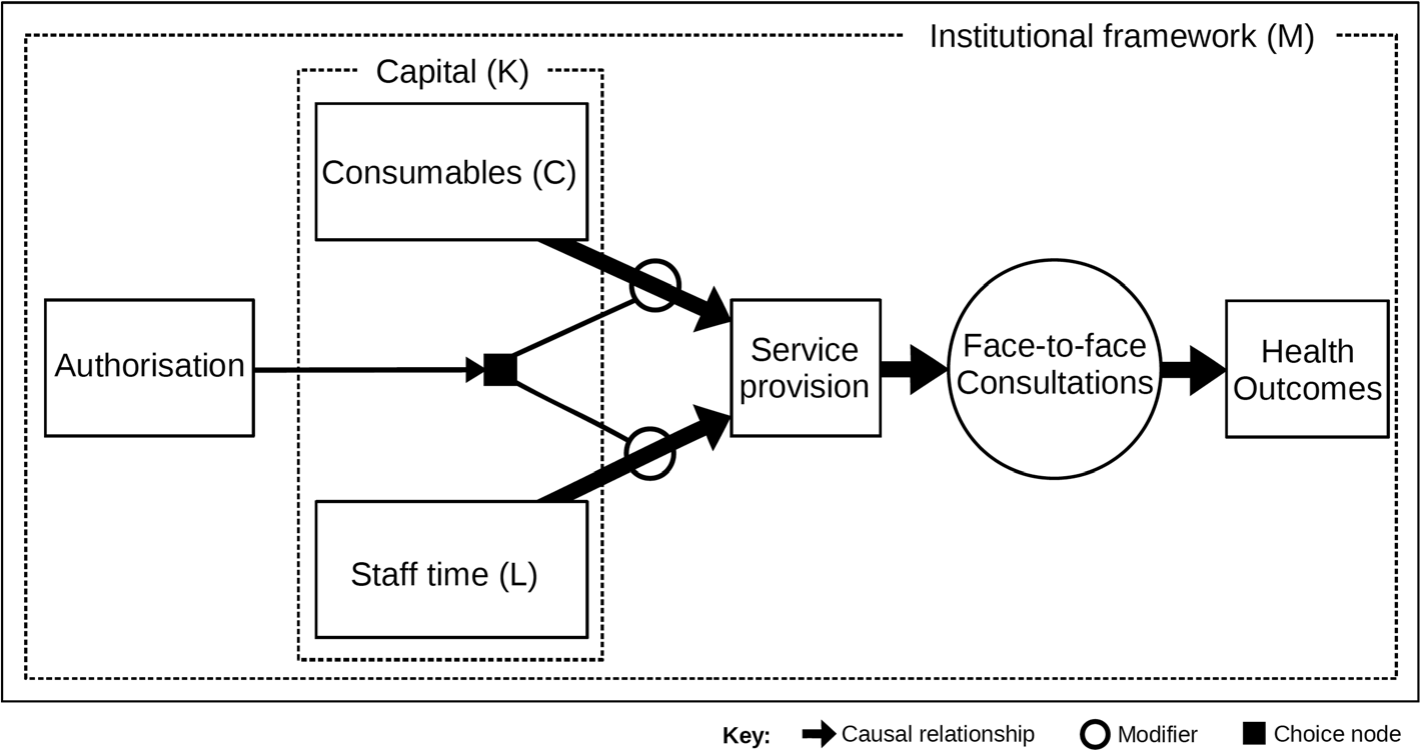
Manual pharmaceutical care

Pharmaceutical care has been adopted internationally as an appropriate model of pharmacy practice. The board of the Pharmaceutical Care Network Europe suggests that ‘pharmaceutical care’ be defined as the ‘pharmacist’s contribution to the care of individuals to optimise medicines use and improve health outcomes’ [24]. Figure 2 reflects this definition. Starting from the left, needed pharmaceutical care services are authorised by the relevant payer, which triggers the choice to expend staff time (usually a qualified pharmacist, but can include other personnel) and related consumables (that is, the materials required to perform a medicines review, for example) to provide the requested service [25]. The amount of labour and consumables expended depends upon the level of capital expenditure (for instance, spending on decision support software). As pharmaceutical care is usually provided as a professional service, the diagram includes a circle that represents the face-to-face interaction between pharmacists and patients and consultation with physicians. If personal consultations with either or both are successful, the advice provided can directly improve patient health outcomes, which is the desired outcome of this philosophy of practice. Finally, pharmaceutical care activities always occur within a governing institutional framework.

## Productivity

‘Productivity’ may be defined as ‘the rate at which labour inputs produce specific production outputs’. The relationship between these two variables may be represented by the production function formula *Y* = *f*(*L*), where Y is an output and L is the input of labour. This relationship is represented diagrammatically for dispensing and pharmaceutical care in the sub-sections below. We assume that staff skills are part of the relationship *f*(L) and that better skilled workforces will produce higher levels of Y with the same labour inputs.

### Dispensing productivity

**Fig. 3:**
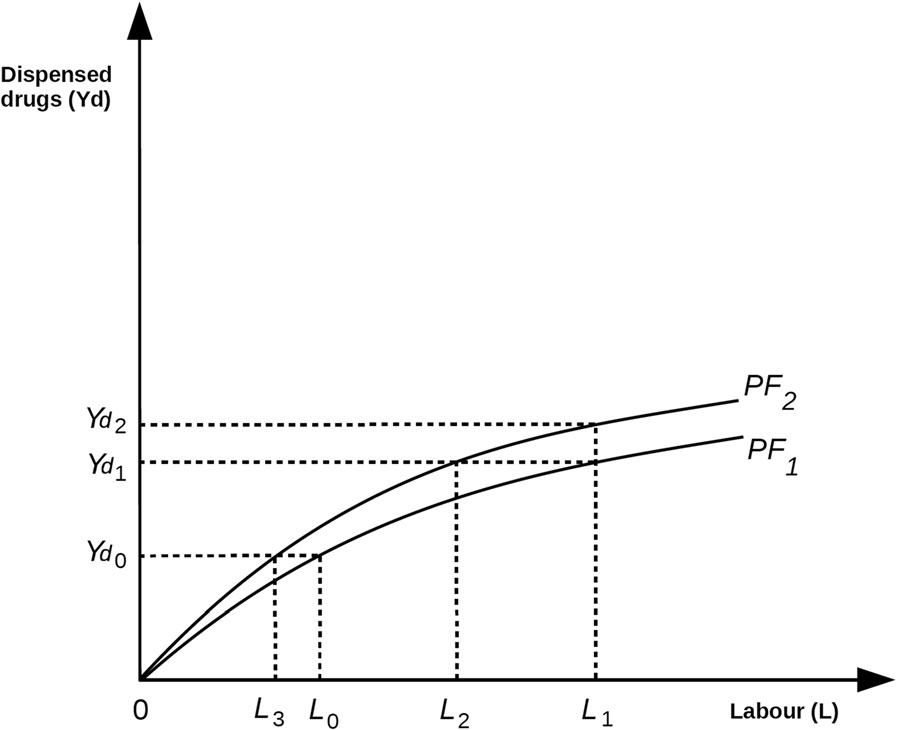
The dispensing production function

Figure 3 shows the production relationship space for labour inputs and the output of medicines dispensed, *Yd*. The curve PF_1_ shows the relationship between *L* (pharmacist and technician time) and the number of items supplied. Its shape suggests ‘diminishing marginal returns’ in the sense that, as more labour is employed, more items are dispensed, but workers become less productive per unit completed. At the level of labour input *L*_0_ the dispensing volume supplied is *Yd*_0_. If more labour is utilised, shifting the input from *L*_0_ to *L*_1_, then output increases from *Yd*_0_ to *Yd*_1_. This is called a ‘shift along the production function’. If the rate of productivity shown by PF_1_ does not increase, then more labour must be expended if dispensing volumes rise. If workers become more productive (perhaps due to better training), then a ‘shift in the production function’ may occur from PF_1_ to PF_2_. As a result, labour input L_1_ can now produce *Yd*_2_. If the production function shifts from PF_1_ to PF_2_ but dispensing volumes do not rise, then *Yd*_0_ may be produced with *L*_3_ labour and *Yd*_1_ with *L*_2_. Figure 3 therefore shows that dispensing volumes may be increased by (i) raising labour inputs, (ii) improving productivity or (iii) a combination of both.

### Pharmaceutical care productivity

**Fig. 4:**
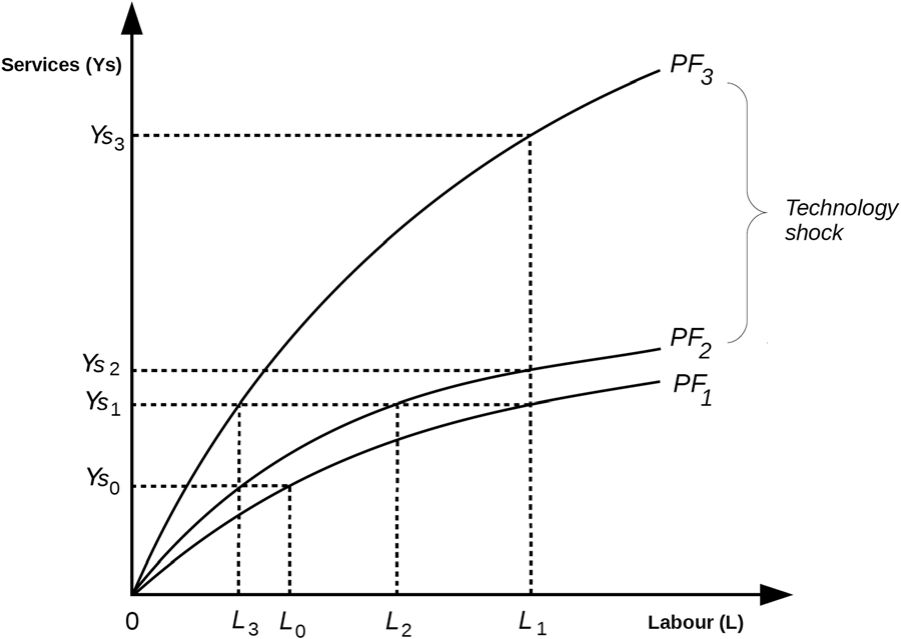
The pharmaceutical care production function

Although pharmaceutical care pharmacists are service providers, the primary purpose of their work is not to deliver consultations, but to maximise the quantum of health gain they secure. In other words, they focus on patient outcomes not service outputs. Nevertheless, the economic concept of productivity measures physical outputs not subsequent benefits secured by consumers. Therefore, Fig. 4 shows the relationship between labour inputs and pharmaceutical care outputs measured in terms of services delivered, *Ys*, not health gains. Using the normal economic assumption of diminishing marginal returns, PF_1_ shows the initial relationship between *L* and *Ys*. At the level of labour input *L*_0_ the production of services is *Ys*_0_. If more labour is expended at *L*_1_, then output increases to *Ys*_1_. As workers become more productive (for instance, due to education or training), the production function shifts from PF_1_ to PF_2_. With the new production function, *L*_1_ can produce *Ys*_2_. Although all productivity gains are important, the improvement from *Ys*_1_ to *Ys*_2_ is relatively marginal. In response, one way of securing a substantial improvement in productivity is the introduction of an innovation that creates a ‘technology shock’. For instance, online telecare facilities may greatly increase the number of services that pharmacists in rural areas may complete per hour [26, 27]. The effects of such a shock are shown by PF_3_, which is a major increase in productivity compared to PF_2_. With this relatively substantial jump, *L*_1_ can now produce *Ys*_3_. Alternatively, if *Ys*_1_ services are produced, PF_1_ requires the input of *L*_1_ labour, PF_2_ requires *L*_2_ inputs and PF_3_ requires *L*_3._ These responses demonstrate that the technology shock is clearly beneficial compared with the original level of productivity, PF_1_, or the incremental gain, PF_2_. Therefore, we may conclude that technological innovation can be vitally important to pharmacy practice productivity.

## Technology

‘Technology’ may be defined as the ‘dynamic clustering of techniques, methods, skills and processes used in the production of goods or services or in the achievement of outcomes that deliver desired benefits for consumers’. Our framework represents the different ways technology and education can impact upon pharmacy practice and productivity.

### Prescribing-dispensing technologies

**Fig. 5:**
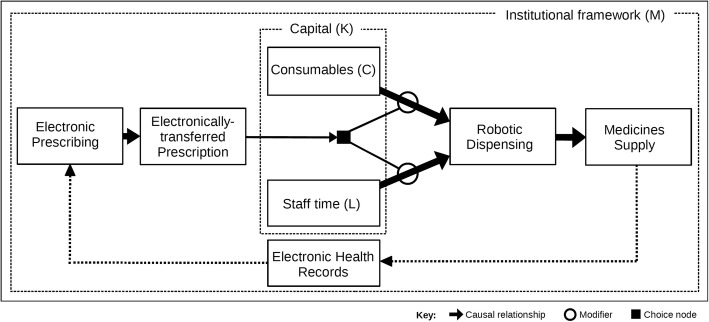
Automated dispensing process

Figure 5 is a conceptualisation of an automated dispensing process. Automated systems connect prescribers to the dispensing process through a series of inter-related technologies. To successfully integrate, the process requires interoperability between its separate data sources and software programmes. In this automated system, demand is generated by prescribers using electronic prescribing software and is received in the dispensary as electronically transferred prescriptions. These electronic requests trigger the use of consumables and staff time, which are either released manually (by an authorised decision-maker) or automatically (within the system itself). In our schema, requested drugs are dispensed robotically, which may involve staff resources when medicines are checked and handed-over to patients. The level of human input expended throughout the process will depend upon the degree of automation, which (in turn) depends upon the size of capital expenditure. As the diagram shows, details of medicines supplied are recorded on electronic health records, which are available for prescribers to review. The prescribing-dispensing process is governed by local institutions that have special laws, regulations, policies, rules, conventions, ethics and norms, which also legislate the use of health technologies and patient data. As the automated process covers both drug choice and supply, we refer to the technologies harnessed in this system as prescribing-dispensing technologies.

### Pharmaceutical care technologies

**Fig. 6:**
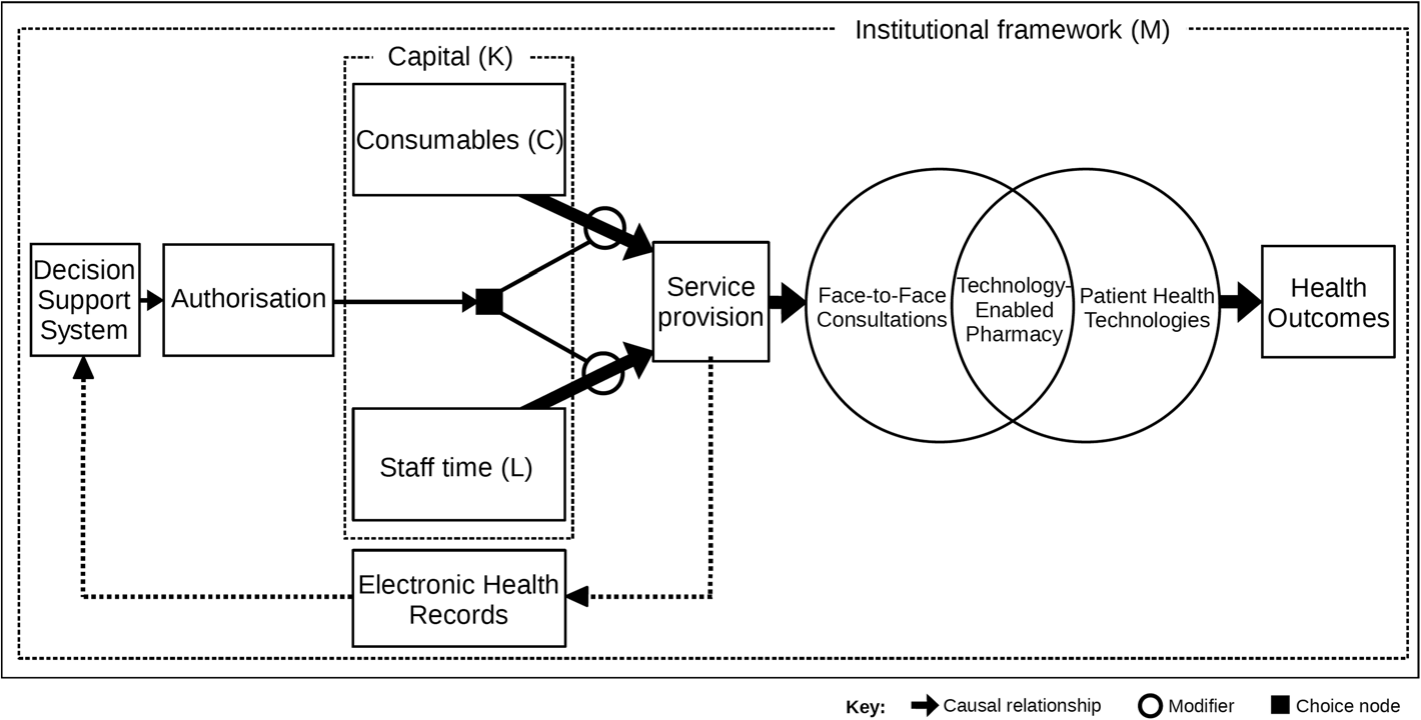
Technology-enabled pharmaceutical care

Figure 6 suggests how a fully integrated, technology-enabled pharmaceutical care process could be structured. The diagram is more ‘normative’ (that is, ‘what should be’) than ‘positive’ (that is, ‘what is’) because few systems of this type yet exist. Starting on the left, decision support software identifies patients requiring a medicine service, which is authorised before being sent as a service request to a providing pharmacist. The subsequent provision expends resources, primarily the expenditure of pharmacist time during face-to-face or phone consultations. During their interactions with patients and prescribers, the use of pharmaceutical care technologies enables pharmacists to be more effective and efficient at service provision. Alongside technology-assisted, face-to-face consultations, patients can adopt their own health technologies (such as mobile health apps) to help manage their own health. Our framework, therefore, differentiates between ‘technology-enabled pharmacy’ and personalised health technologies. The former is an approach to improving the effectiveness and efficiency of service offerings. The latter are sold by manufacturers directly to patients to monitor and to help improve their own health. Working in partnership, the combination of face-to-face consultations with pharmacy and patient technologies could result in better health outcomes, if the circumstances are favourable. To help improve care choices, data (including services provided, technologies harnessed and patient health information) are collected in electronic health records and fed into the appropriate decision support systems. The whole process of providing professional services using ‘pharmaceutical care technologies’ is governed by local institutional frames, suggesting that activities may be structured differently in different places and countries.

## Discussion

The conceptual framework presented here suggests that pharmacy practice and technology are often intimately interconnected. Production processes for dispensing and pharmaceutical care adopt technologies specifically designed to secure their required outputs of, respectively, medicines supplied and services provided.

### Technology-enabled pharmacy

In 2009, the ASHP Section of Pharmacy Informatics and Technology reported that pharmacy practice, especially in the acute sector, had remained unchanged for over 30 years [28]. In response, the section argued that the profession should focus less on drug distribution and more on medicines use. This requires the adoption of new models of practice and supporting technologies that enable pharmacists to (i) reduce their input into the dispensing process, (ii) improve their performance at providing professional services and (iii) secure better patient outcomes [29]. In today’s environment, these reforms could be achieved as follows. First, pharmacist involvement in dispensing could be reduced by switching to more automated processes, thus freeing time for other activities [30]. For example, time would be available to counsel patients on the proper use of their medicines. Next, innovations could be adopted that enable pharmacists to provide their services more efficiently. For instance, decision-support systems could improve the screening of prescriptions and select patients suitable for pharmaceutical care interventions; they can also be embedded in computer prescriber order entry systems to alert prescribers to potential errors when treatment decisions are made, preventing errors proactively [31]. Finally, pharmacists could co-produce better health outcomes by incorporating patient health technologies into their work. For example, by having patients track and trend biomarkers such as blood glucose in diabetics, pharmacists can better monitor treatment outcomes. In addition, utilising digital health and prescribing dispensing technologies especially in low-resource settings would potentially positively impact service and health outcomes; already, existing services that improve medication adherence and access to medicines such as home delivery services are being improved by technological applications that can help patients order and track their deliveries. If these significant improvements are rigorously pursued, future opportunities and boundaries of pharmacy practice could be defined by emerging technologies rather than by the profession’s historically defined roles [32].

As a caveat, the application of technology may threaten a current pharmacy workforce by eliminating some tasks currently performed by pharmacists. There is also the implication that new skills may be needed in the current and future pharmacy workforce to assume the new roles that technology has the potential to enable. However, this framework does not address the issue of workforce directly. In keeping with the mainstream economics approach, we treat pharmacists as an input (L) that is homogeneous and interchangeable. Measuring the he response of an existing labour force to changes in production is beyond the scope of this framework at present. In many cases, an existing workforce may be profoundly threatened by technological change and may not be compliant or positive to implementation, which creates a serious threat to success. As an economic framework, our approach does not encompass such matters. Nevertheless, we acknowledge that the self-interest of the existing workforce is important subject for further study, perhaps through implementation science or sociological studies.

### Blended pharmacy practice

**Fig. 7:**
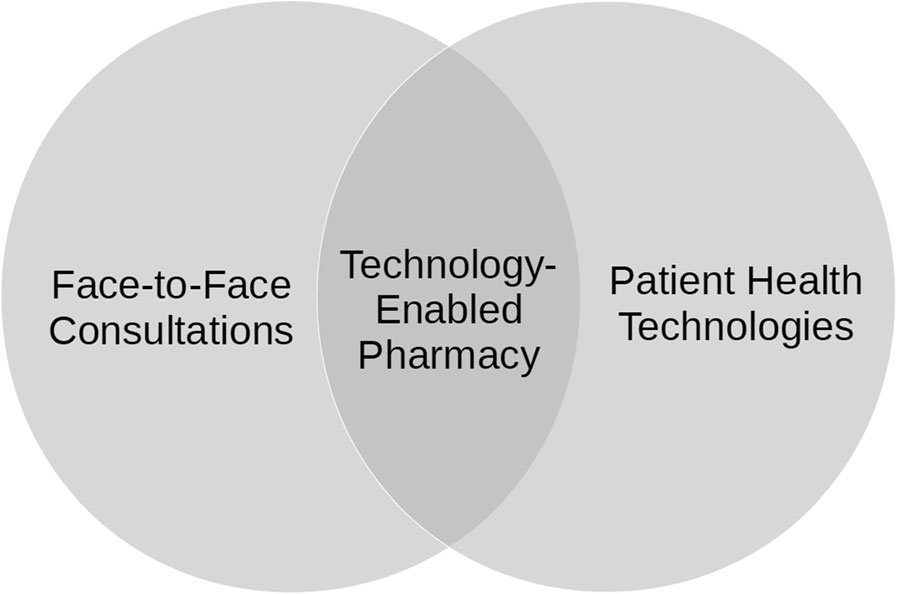
Blended pharmacy practice

At the core of our conceptual framework is the assumption that technology-enabled practice differs significantly from traditional practice methods [33]. Based upon this belief, we argue that improvements in productivity may be achieved by (i) switching to technology-enabled practice, (ii) incremental changes with existing technologies (for instance, due to re-training) and (iii) technology shocks (such as the introduction of a radically better systems for delivering clinical outcomes). Despite the possibilities that innovation offers, the current reality is that most dispensing and pharmaceutical care activities are not technologically advanced, with the slow adoption of technologies of most types. This is not atypical given the insight gained from the study of the diffusion of innovation that shows a time frame of decades for new ideas to be adopted [34] situation may be described using Fig. 7. On the left, some face-to-face consultations are delivered manually, with no technological support whatsoever. In the middle, specific technologies exist that may be used to directly enable professional working. On the right, patients may purchase their own health technologies that, in suitable circumstances, supplement the preceding approaches to service provision. In response to this mixture of manual and technological processes, we suggest that the term ‘blended pharmacy practice’ should be used to describe the profession’s working practices in the twenty-first century.

### Education and technology

In 2017, FIP published a technical report, Transforming Pharmacy and Pharmaceutical Sciences Education in the Context of Workforce Development [8]. The work acknowledges that, in response to the redefining concept of pharmaceutical care, pharmacy practice has undergone a progressive shift from being primarily product-oriented (in terms of medicines supply) to becoming more patient-oriented (in terms of generating health outcomes) [35]. With the aim of enhancing standards worldwide, the document contains 67 statements on Pharmacy and Pharmaceutical Sciences Education (‘the Nanjing Statements’) that describe an envisioned future for professional education and training. Nanjing Statement number 7.3 states that ‘policies and procedures support regular review of the curriculum and allow developments in the curriculum to take place in a timely manner so as to keep up with the changes in the profession, technology and society’. This statement clearly acknowledges the intimate connection between pharmacy practice, technology and patient behaviours. To support workforce development, we suggest that ‘blended practice’ should now become a core concept in pharmacy education, both for the existing and future pharmacy workforce worldwide.

## Conclusions

Technology has the potential to shape the nature and values of pharmacy practice, with implications for professional roles, power, jurisdictions and boundaries [36]. Our novel framework is designed to facilitate policy, education and research by providing an analytical approach to service delivery. By using our approach, the profession could develop examples of good practice in both developed and developing countries worldwide. We believe that this important policy agenda should be supported by the following research activities: (i) further study of how the concept of technology applies to pharmacy practice, (ii) scoping reviews of the literature on technology-enablement in dispensing and pharmaceutical care and (iii) high-quality qualitative and quantitative evaluations of new technologies employed in a pharmacy setting. Finally, we conclude that the profession should acknowledge the importance of technology in determining the techniques, methods, skills and processes embedded in dispensing and pharmaceutical care practice. Addressing problems with medication use with the development of a pharmaceutical workforce that is sufficient in quantity and competence is a long-term issue. As companion strategy, utilising technology to make the healthcare system more efficient such that there is less demand on workforce can be a shorter path to improvement. Education and training programmes will be essential to prepare the current and future pharmacy workforce to realise the potential benefits of technology to improve the responsible use of medicines.
